# Advantages and limitations of shot-gun proteomic analyses on Arabidopsis plants with altered MAPK signaling

**DOI:** 10.3389/fpls.2015.00107

**Published:** 2015-02-25

**Authors:** Tomáš Takáč, Jozef Šamaj

**Affiliations:** Department of Cell Biology, Faculty of Science, Centre of the Region Haná for Biotechnological and Agricultural Research, Palacký UniversityOlomouc, Czech Republic

**Keywords:** mitogen-activated protein kinase (MAPK), signaling, proteomics, phosphoproteomics, shot-gun proteomics, validation

## Introduction

The molecular mechanism of signal transduction by mitogen-activated protein kinases (MAPKs) in plants is an intensive research field of contemporary plant biology. Signaling in plants involves perception of changing environment and the intracellular transduction of such generated information leading to the activation of adaptive mechanisms or programmed cell death, as it is in the case of hypersensitive response to some biotrophic pathogen attack. At the molecular level, distinct signaling pathways, such as MAPK modules, involve reversible phosphorylation, enzymatic activation, protein-protein interaction and finally transcriptional activation via nuclear-resident transcription factors or cell reorganization via regulation of cytoplasmic or plasma membrane-associated proteins. All these processes are strictly regulated and subcellularly compartmentalized.

Modern OMICS technologies encompassing transcriptomic (Krysan et al., 2002; Frei dit Frey et al., 2014), metabolomic (Lassowskat et al., 2014), proteomic (Miles et al., 2009; Conroy et al., 2013; Lassowskat et al., 2014; Takáč et al., 2014) and phosphoproteomic (Hoehenwarter et al., 2013; Lassowskat et al., 2014) approaches have been employed to gain functional information on plant signaling. The main challenges of advanced (phospho)proteomic studies are: reliable identification of all signaling constituents of a particular MAPK pathway as well as identification and validation of respective target proteins. In this opinion article, we aim to briefly evaluate benefits and pitfalls of various proteomic approaches that are used to study MAPK signaling in plants. We emphasize on the advantages of comparative shot-gun proteomic analyses of Arabidopsis mutants and transgenic plants with altered MAPK signaling. The importance of critical data validation and interpretation is also discussed.

## Proteomic approaches on MAPK signaling

Modern proteomics offers a variety of approaches to study cell signaling events in living organisms (Jørgensen and Locard-Paulet, 2012). They have been quite rarely used also for investigation of molecular mechanisms in plant MAPK signaling (Table 1). Nevertheless, these studies substantially helped to better describe complex processes accompanied with signaling and to find new kinase targets regulated by reversible phosphorylation.

**Table 1:** **Overview of proteomic approaches on MAPK signaling in plants**.

**Proteomic method**	**Species, genotype**	**Sample**	**Key findings**	**References**
Protein and peptide microarray-based proteomic analysis	*Arabidopsis thaliana*, protein microarray derived from an inflorescence meristem cDNA expression library incubated with purified MPK3 and MPK6 in the presence of [γ^−33^P]ATP	Inflorescence meristem	Identification of 48 potential substrates of MPK3 and 39 of MPK6 26 common substrates of both MPK3 and MPK6	Feilner et al., 2005
	*Arabidopsis thaliana*, protein microarray prepared by purification of 2158 proteins expressed in *Nicothiana benthamiana* incubated with *in planta* activated 10 different MAPKs	Arabidopsis proteins, tissue not specified	Identification of 570 MAPK substrates 290 putative substrates were phosphorylated only by one MAPK	Popescu et al., 2009
	*Solanum lycopersicum*, 976 synthetic peptides containing phosphorylation sites for different types of human kinases incubated with recombinant LeMPK1, LeMPK2, LeMPK3 and analyzed using kinase assay	Synthetic peptides	30% of peptides phosphorylated by all three LeMPKs Overlapping but also different phosphorylation preferences were found	Stulemeijer et al., 2007
Total proteome dissection of mutants and transgenic plants with genetically manipulated MAPKs	*Arabidopsis thaliana, anp2anp3* double mutant	Seedlings	25 differentially abundant proteins Constitutive activation of oxidative stress defense Increased resistance to oxidative stress in *anp2anp3* mutant	Takáč et al., 2014
	*Arabidopsis thaliana*, SIMKK-YFP overexpressing plants	Roots	22 differentially abundant proteins constitutive decreased abundance of key proteins involved in salt stress tolerance in SIMKK-YFP overexpressing plants	Ovečka et al., 2014
	*Arabidopsis thaliana*, LOF (*yda1)* and GOF (Δ*Nyda*) mutants of YODA (MAP3K4)	Seedlings	122 differentially abundant proteins in *yda1* 108 differentially abundant proteins in Δ*Nyda* increased abundance of proteins important for auxin biosynthesis	Smékalová et al., 2014
	*Arabidopsis thaliana*, RNAi-AtMPK6 plants treated with ozone	Rosette leaves	14 differentially abundant proteins in untreated transgenic line compared to Wt 31 differentially abundant proteins in ozone-treated transgenic line compared to Wt Increased abundance of proteins of antioxidant defense	Miles et al., 2009
	*Arabidopsis thaliana*, MKK1 knock out mutant exposed to salt stress	Leaf	5 differentially abundant proteins Increased abundance of proteins involved in energy production in the MKK1 knock out plants	Conroy et al., 2013
	*Arabidopsis thaliana*, wild type*, mpk3* and *mpk6* mutants expressing constitutive active MKK5 from *Petroselinum crispum*, under the control of a dexamethasone inducible promoter	Leaf	Upregulation of proteins of primary metabolism, tryptophan biosynthesis, and glucosinolate metabolism; downregulation of proteins involved in translation in transformed *mpk3* plants	Lassowskat et al., 2014
Phosphoproteomics on mutants and transgenic plants with genetically manipulated MAPKs	*Arabidopsis thaliana*, plants expressing constitutively active *MEK2^DD^* from *Nicotiana tabacum* under the control of the dexamethasone-inducible GVG promoter	Seedlings	733 phosphosites (372 novel) in activated mutants 382 phosphoproteins 141 MPK substrate candidates	Hoehenwarter et al., 2013
	*Arabidopsis thaliana*, plants expressing a constitutively-active as well as inactive variant of *MKK5* from *Petroselinum crispum*, under the control of a dexamethasone-inducible promoter	Leaf	Identification of early and late phosphorylation targets 144 phosphoproteins with altered abundance after 4 h of DEX treatment involving proteins involved in vesicle-mediated and nucleocytoplasmic transport 729 phosphoproteins with altered abundance after 8 h of DEX treatment, including proteins involved in embryo development, stress and immune responses	Lassowskat et al., 2014
Phosphoproteomics based on ^15^N stable isotope metabolic labeling	*Arabidopsis thaliana*, ethylene-treated wild type, ethylene-overly-sensitive loss-of-function mutant *rcn1-1* and loss-of-function ethylene signaling *ctr1-1* mutant, deficient in MAP3K CTR1	Seedlings	Identification of ethylene-regulated phosphosites and phosphoproteins acting in both CTR1 kinase- and PP2A phosphatase-mediated phosphor-relay pathways	Yang et al., 2013

Targeted phosphoproteomics is a powerful tool for the identification of putative downstream substrates of MAPKs, but also for mapping of phosphorylation sites detected upon activation or inactivation of particular MAPK pathways (de la Fuente van Bentem et al., 2008; Hoehenwarter et al., 2013; Lassowskat et al., 2014). Diverse phosphoproteomic approaches have been applied, mostly employing genetically modified kinase expression systems. For example, a recent phosphoproteomic study of transgenic *Arabidopsis thaliana* plants harboring a gene encoding a constitutively active *MEK^DD^* from *Nicotiana tabacum* (NtMEK2^DD^) expressed under the control of the dexamethasone (DEX)-inducible GVG promoter identified 141 putative MAPK substrates with the help of complementary enrichment of phosphoproteins and consecutive phosphopeptide enrichment (Hoehenwarter et al., 2013). In a similar approach, early and late putative substrates of MPK3 and MPK6 were identified by phosphoproteomics performed on *Arabidopsis thaliana* plants transformed with a constitutively-active variant of *MKK5* from *Petroselinum crispum*, expressed under the control of a DEX-inducible promoter (Lassowskat et al., 2014). The expression of heterologous MAP2Ks in Arabidopsis might result in the identification of somehow unspecific or indirect targets. This problem might be solved by validation of putative MAPK substrates by both bioinformatic and independent analytical methods.

The phosphoproteomics following stable isotope labeling in Arabidopsis (SILIA) efficiently elucidated a protein complex containing ethylene receptor ETR1 and constitutive triple response 1 (CTR1) MAP3K (Yang et al., 2013). Using the loss of function ethylene response Arabidopsis mutant *ctr1-1*, novel CTR1 targets involved in ethylene response were identified including calcium-sensing receptor and plastidal transcriptionally active protein.

Previously, microarray containing 1690 proteins from Arabidopsis was generated and incubated with MAPKs in the presence of [γ^−33^P]ATP. Quantitative evaluation of radioactive signals identified 48 potential substrates of MPK3 and 39 substrates of MPK6 (Feilner et al., 2005). A more extended protein microarray-based approach for MAPK phosphorylation target analysis was published by Popescu and coworkers (Popescu et al., 2009). First, purified wild type MAPKs (MPK1–MPK8, MPK10, and MPK16) were tested by *in vitro* phosphorylation assays for their activation by certain MAPKKs co-expressed in *Nicothiana benthamiana* leaves. Subsequently, 2156 proteins were placed on protein microarray and probed by above MAPKs. This approach allowed the identification of new signaling modules composed of MAPKKs, MAPKs and their target proteins.

Peptide microarray analysis based on screening of hundreds of synthetic peptides containing experimentally verified phosphorylation sites for different types of human kinases was used to discriminate between *Solanum lycopersicum* LeMPK1, LeMPK2, and LeMPK3 (Stulemeijer et al., 2007). The incubation of peptide microarrays with recombinant LeMPK1, LeMPK2, and LeMPK3 in the presence of [γ^−33^P]ATP was followed by *in vitro* kinase assay which displayed both similar, but also different peptides phosphorylated by these MAPKs.

In our opinion, another promising tool for MAPK substrate identification was provided by recent study, which used a library of 377 synthetic peptides, representing previously identified phosphorylation sites in developing seeds of *Arabidopsis thaliana* and *Brassica napus*, for screening with purified kinases while the resulting phospho-sites were analyzed subsequently by mass spectrometry (Ahsan et al., 2013). It is of great value that all these diverse phosphoproteomic approaches helped to identify new putative targets of MAPKs. However, in our view they lack the capability of shot-gun comparative proteomics to identify mid- and long-term complex protein profiles and networks associated with the transcriptional impact of deregulation of MAPK pathways, as this seems to be often the case in the mutant and overexpressor plant lines (e.g., Krysan et al., 2002). We consider this point quite important because changed complex protein profiles revealed by shot-gun proteomics might be closely linked to phenotypes of respective mutant and transgenic lines, including their characteristics such as modified stress responses or developmental defects.

## Benefits and weaknesses of comparative shot-gun proteomics on arabidopsis MAPK mutants and transgenic overexpression plants

Complex molecular processes affected by deregulation of MAPK signaling cascades *in planta* are often focused on transcriptomic studies (Frei dit Frey et al., 2014) while global protein changes are still underestimated. Since conditionally-modulated transcript levels can be rectified at the protein level, it is of essential importance to perform proteomics rather than speculate on protein abundance based on transcriptomic data (Gygi et al., 1999). Shot-gun proteomic analyses on mutant and transgenic plants with genetically modified MAPKs and their upstream activators such as MAP2Ks and MAP3Ks provide a very useful proteome readout and quite unique information because they might not be fully consistent with transcriptomic data from the same plant lines (e.g., Krysan et al., 2002 cf. Takáč et al., 2014). Few studies used shot-gun proteomic approaches to better explain stress-related and developmental phenotypes in Arabidopsis mutants and transgenic lines with altered MAPKs expression. One earlier study employing ICAT (isotope-coded affinity tag) quantitative proteomic analysis on *MPK6 RNAi* transgenic Arabidopsis plants unveiled molecular mechanisms accompanying response to ozone fumigation in relation to MPK6 downregulation (Miles et al., 2009). The redox response pathway was different between untreated plants with suppressed MPK6 expression and the corresponding control wild type plants. After ozone treatment, a more pronounced antioxidant defense was observed in plants with downregulated MPK6 (Miles et al., 2009). Recently, the two-dimensional LC MS/MS analysis of Arabidopsis double mutant deficient in two redundant MAP3Ks called ANP2 and ANP3 revealed constitutive upregulation of a protein functional network involved in oxidative stress response in the double mutant *anp2anp3* plants (Takáč et al., 2014). This suggested an increased resistance of the *anp2anp3* mutant against oxidative stress which was corroborated by showing increased viability of this mutant growing on paraquat-supplemented medium. The activation of a protein network consisting of superoxide dismutase isoforms, chaperonin 20 and enzymes of the ascorbate-glutathione cycle was validated through detailed biochemical, physiological and histochemical analyses showing connection of proteomic data with resilience of the above mutant to oxidative stress. Importantly, this shot-gun proteomic study uncovered a previously unknown function of ANP2 and ANP3 in Arabidopsis antioxidant defense.

Next, we reported that the heterologous overexpression of alfalfa SIMKK-YFP leads to salt hypersensitivity of Arabidopsis seedlings. The proteomic analysis showed decreased levels of several key proteins involved in salt tolerance in the roots of these SIMKK-overexpressor transgenic plants (Ovečka et al., 2014). Thus, the overexpression of SIMKK repressed the levels of catalase, peroxiredoxin, glutathione S-transferase, nucleoside diphosphate kinase 1, endoplasmic reticulum luminal-binding protein 2, and finally those of plasma membrane aquaporins. Downregulation of these proteins most likely contributed to the increased salt sensitivity of transgenic Arabidopsis seedlings overexpressing SIMKK (Ovečka et al., 2014).

In addition to the analysis of stress responses, shot-gun proteomic analyses on mutant and transgenic plants might help to explain unique growth or developmental phenotypes regulated by members of particular MAPK pathway. Recently, we have found that both inactivation and constitutive activation of MAP3K called YODA caused pronounced root phenotypes in Arabidopsis (Smékalová et al., 2014). Analysis of endogenous auxin levels showed that these phenotypes might be related to increased IAA production in respective *yda1* and Δ*Nyda* mutants. Again, shot-gun proteomics provided insights to the molecular mechanism underlying auxin overproduction by detection of elevated levels of proteins involved in auxin biosynthesis such as tryptophan synthase in Δ*Nyda* and nitrilases in both mutants (Smékalová et al., 2014).

Finally, a recent elegant study used integrated metabolomics, shot-gun proteomics and phosphoproteomics approaches on leaves of Arabidopsis Col-0 ecotype as well as *mpk3* and *mpk6* mutants, all expressing DEX-inducible constitutively active *Petroselinum crispum* MKK5 (MKK5^*DD*^). Heterologous MKK5^*DD*^ expression activated MPK3 and MPK6 at equal levels in Col-0 plants. The induction of MKK5^*DD*^ in the *mpk6* mutant resulted in more robust proteome changes compared to *mpk3*. In this case, shot-gun proteomic data were in positive correlation with metabolomic as well as phosphoproteomic analyses (Lassowskat et al., 2014).

All these shot-gun proteomic studies helped to identify new molecular processes linked to MAPK pathways. This capability could be assigned to phosphoproteomics as well, as it was shown for example for nitrogen metabolism, control of circadian clock and phototropism (Hoehenwarter et al., 2013), and for biosynthesis of antimicrobial defense substances (Lassowskat et al., 2014).

Proteomics on total protein extract has technically limited capability for identification of all molecular mechanisms connected with particular MAPK pathway. These limitations arise from limited proteome resolution especially in terms of detection of low abundant proteins and inability of this approach to resolve phosphorylation events. Nevertheless, we consider these studies feasible in order to elucidate molecular networks composed from abundant proteins, including those involved in antioxidant defense (Miles et al., 2009; Takáč et al., 2014) or stress response (Ovečka et al., 2014). In our view shot-gun proteomics can also provide important initial insights into new biological processes regulated by MAPKs, which should be later investigated by targeted application of genetic and cell biological methods. In ideal case, general shot-gun differential proteomics may be perhaps quite efficiently complemented by targeted phosphoproteomic (Lassowskat et al., 2014). In this respect, dual-enrichment techniques (Hoehenwarter et al., 2013; Lassowskat et al., 2014) might represent a crucial tool in order to overcome the limitation of the large dynamic range of protein abundance in shot-gun proteomic approaches.

## Evaluation and validation of proteomic data

The complexity of proteomic analysis and the high inter-replicate variability of the proteomic data necessitate proper independent validation methods to confirm proteomic results. This significantly increases the biological value of the proteomic analysis. For example, protein abundance might be verified using immunoblotting with monospecific primary antibodies (Takáč et al., 2014). As an alternative, immunolocalization of protein in intact respective tissue have been also employed (Takáč et al., 2013) and provide useful information about spatial protein localization. The live cell microscopy imaging using fluorescently tagged proteins may be also used to study protein localization (Takáč et al., 2012). Nevertheless, to study abundance of fluorescently tagged protein of interest it should be expressed under the control of its own promoter in the respective mutant background (Smékalová et al., 2014). Transgenic mutant lines with such construct have to show rescue of the mutant phenotype which is considered as a proof of construct functionality. Subsequently, such rescued mutants bearing tagged protein can be used for isolation of functional protein complexes and MS analysis. To validate enzymatic activity, specific spectrophotometric measurements and/or activity staining on native polyacrylamide gels might be used (Takáč et al., 2014). The validation of protein phosphorylation is possible via kinase assays (Feilner et al., 2005; Yang et al., 2013; Lassowskat et al., 2014), by using Phos-tag technology (Kinoshita et al., 2006; Komis et al., 2014) or by isoelectric focusing combined with immunoblotting (Anderson and Peck, 2008). Protein-protein interactions can be tested by BIFC or co-immunoprecipitation assays (Singh et al., 2012).

The efficiency of shot-gun proteomics for the exploration of MAPK signaling pathways could be significantly increased by bioinformatic evaluation of proteomic data. There are several commercial software and web-based applications providing various analyses including functional categorization of differentially abundant proteins (Conesa and Götz, 2008), clustering of commonly expressed proteins (de Hoon et al., 2004), metabolic pathway analysis tools (Iersel et al., 2008), applications summarizing protein-protein interactions (Jensen et al., 2009), coexpressing protein candidates (Obayashi and Kinoshita, 2010), tools for prediction of protein localization (Horton et al., 2007), detection of signaling peptides (Petersen et al., 2011), search for most abundant sequence motifs (Chou and Schwartz, 2011) and others.

## Goal-oriented approaches and complementarity in complex cell signaling analysis

As explained above, the high throughput analyses of MAPK signaling such as transcriptomics, proteomics and phosphoproteomics are not always necessarily converging to a common output. They have, at least partially, different scopes and their results might be either complementary or contradictory (Gygi et al., 1999). Protein array surveys identify a list of putative substrates of MAPKs but outside of a physiological context. In this sense, phosphoproteomic analyses will decipher phosphoproteins in MAPK mutants or under conditions triggering MAPK activity but with uncertainty as to whether they truly constitute exclusive MAPK substrates (they might be eventually phosphorylated also by alternative kinases). Finally, proteomics (including shot-gun approaches) has potential to corroborate the transcriptional transactivation role of MAPK signaling and should provide factual data on the protein levels. Although proteomics will not reveal mechanistic insights of MAPK signaling (i.e., identify MAPK substrates) it will provide the landscape of its physiological consequences which can be efficiently followed by targeted biochemical, genetic cell biological and physiological assays.

Altogether, this article summarizes recent evidence that shot-gun proteomic analysis of Arabidopsis mutants and transgenic lines with genetically-manipulated MAPK expression is a reliable method for the detection of some molecular networks accompanying developmental or conditional phenotypes of these mutants and lines. We strongly recommend using proper independent methods, including biochemical, cell biological and phosphoproteomic ones, for validation and corroboration of proteomic data. Such detailed studies may eventually provide new links between MAPK-related changes in protein abundance and direct regulation of cellular processes through MAPK-dependent phosphorylation of target proteins.

### Conflict of interest statement

The authors declare that the research was conducted in the absence of any commercial or financial relationships that could be construed as a potential conflict of interest.
